# The Process of Osteoblastic Infection by *Staphylococcus Aureus*

**DOI:** 10.7150/ijms.45960

**Published:** 2020-05-29

**Authors:** Qiangqiang Wen, Feng Gu, Zhenjiang Sui, Zilong Su, Tiecheng Yu

**Affiliations:** Department of Orthopedics, First Hospital of Jilin University, Changchun 130021, Jilin, China.

**Keywords:** Osteoblastic, Infection, Staphylococcus Aureus, Antimicrobial Peptides

## Abstract

Bone infection is difficult to cure, and relapse frequently occurs, which is a major treatment problem. One of the main reasons for the refractory and recurrent nature of bone infection is that bacteria, such as Staphylococcus aureus (S. aureus), can be internalized into osteoblasts after infecting bone tissue, thereby avoiding attack by the immune system and antibiotics. Understanding how bacteria (such as S. aureus) are internalized into osteoblasts is key to effective treatment. S. aureus is the most common pathogenic bacterium that causes bone infection. This paper reviews the literature, analyzes the specific process of osteoblastic S. aureus infection, and summarizes specific treatment strategies to improve bone infection treatment.

## Introduction

**Figure 1:**
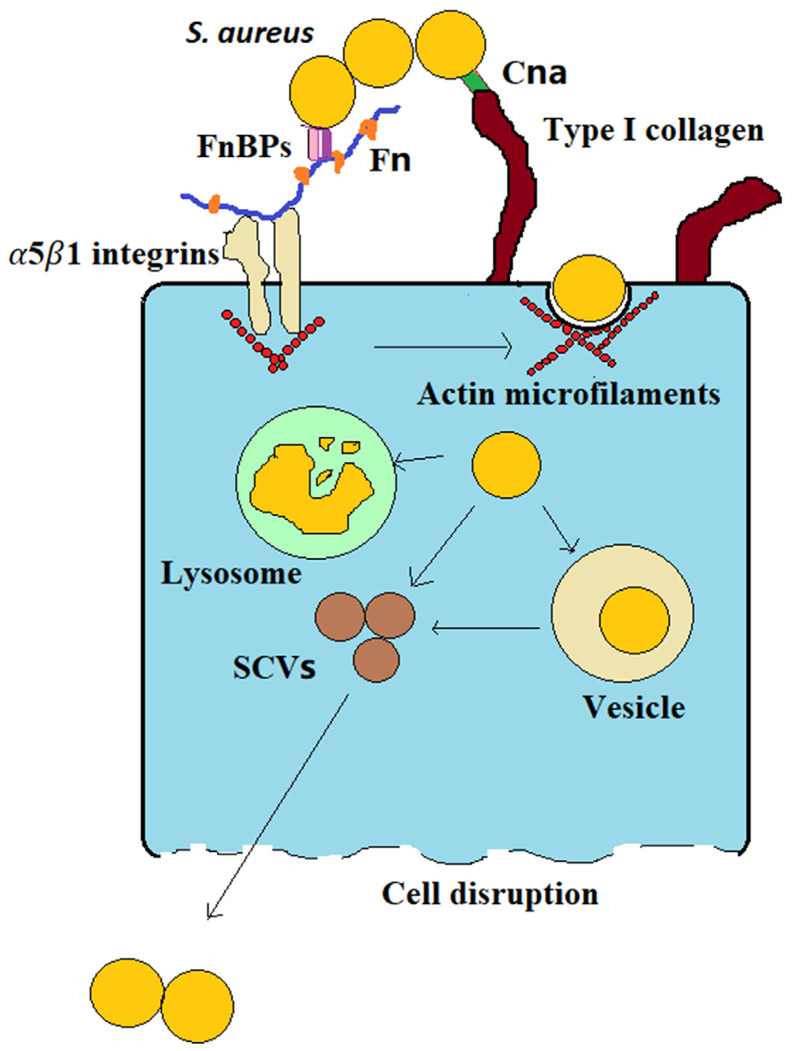
*S. aureus* becomes internalized in osteoblasts.

Bone infection is difficult to cure, frequently relapses, has a high disability rate, and causes great physical trauma and financial burdens to patients. One of the main reasons that bone infections are difficult to cure is that bacteria (such as S. aureus) can invade osteoblasts; in this way, they can evade the body's immune system and protect them from the action of antibiotics. This latter renders them difficult to completely eliminate, leading to recurrent infections 1,2. The infection process can be simply summarized by three stages: (1) S. aureus binds to the bone extracellular matrix (BEM), (2) fibronectin (Fn) receptors mediate S. aureus internalization into osteoblasts, and (3) infected osteoblasts are destroyed (Figure 1). (1) S. aureus binds to the BEM through its cell wall-anchored (CWA) proteins while contacting bone tissue in preparation for subsequent internalization. (2) S. aureus binds to Fn receptors through its surface Fn-binding proteins (FnBPs), and then Fn receptors, such as α5β1 integrins, mediate S. aureus internalization into cells. Upon entering the cell, the bacteria evade the immune system and antibiotics via vesicle escape or small-colony variant (SCV) formation. (3) During the internalization of S. aureus by osteoblasts, osteoblast activity decreases, and apoptosis and death occur. Moreover, osteoclasts are activated, bone resorption increases and bone homeostasis is disrupted. The division of the process of S. aureus osteoblast infection into three stages will be greatly significant for guiding the treatment of bone infection.

## *S. aureus* binding to the BEM

The binding of S. aureus to the extracellular matrix is a key step in its invasion of host cells 3, which is closely related to the pathogenicity of S. aureus. The extracellular matrix, which is produced by osteoblasts, is a site of S. aureus binding. The extracellular matrix includes glycans and proteins such as type I collagen (Cn), bone sialoprotein, osteopontin and Fn. The extracellular matrix can create conditions to promote the accumulation of S. aureus near osteoblasts.

The ability of CWA proteins on the surface of S. aureus to bind components of the extracellular matrix of human bone cells determines whether S. aureus can infect cells. A total of 4 CWA protein types have been identified in S. aureus: the microbial surface component recognizing adhesive matrix molecule (MSCRAMM) family, the near iron transporter (NEAT) motif family, the three-helical bundle family and the G5-E repeat family 4. These CWA proteins have various functions, such as adhering to and invading host cells and tissues and evading the immune response. In the process of S. aureus infection in bone tissue, the Cn adhesin (Cna) protein (a member of the MSCRAMM family) plays an important role because Cn is the main component in the extracellular matrix of bone cells, and the Cna protein mainly plays a role by adhering to Cn^5^. Cna proteins consist of a series of molecular modules, including the N-terminal A domain (consisting of N1, N2, and N3), B repetitive sequence, the cell wall anchoring domain and a short cytoplasmic domain. Cna binds to Cn primarily through a tightly wrapped mechanism (the “collagen hug” mechanism). It is now clear that the N1 and N2 domains in the N-terminal A domain (which has an IgG fold-like structure) and the B repetitive sequence are involved in this mechanism 6,7. The N1 and N2 substructural domains coencapsulate the rope-like structure of Cn and play a major role in this mechanism^6^. In addition to binding to Cn, Cna can bind to the complement protein C1q and laminin (Lam) proteins for adhesion, but the effect is relatively small. In addition, the Cna and C1q complex can also protect the bacteria from the immune system by inhibiting complement system activation through the classical pathway 8.

CWA proteins are essential virulence factors for S. aureus survival during commensal and invasive infections and thus are vaccine targets against S. aureus infection 4. For example, all MSCRAMMs have similar structures, in which two adjacent IgG folding regions mediate their binding to components such as Cn, fibrinogen, or Fn of the host extracellular matrix 4,9, so this folding region can be used as a therapeutic target.

## Fn receptors mediate S. aureus internalization into osteoblasts

S. aureus may survive phagocytosis by neutrophils and macrophages and may even be internalized by osteoblasts 4. The uptake of S. aureus by osteoblasts is mediated by adhesins. The major adhesins are FnBPs 4. FnBPs on the surface of S. aureus make it possible for S. aureus to internalize into osteoblasts via the Fn junction with Fn receptors such as α5β1 integrins 4. Fn is a “bridge” between S. aureus and osteoblasts (Figure 1). This “Fn bridge” enables S. aureus to enter osteoblasts via internalization 10,11. Intracellular frameworks, especially actin microfilaments, are involved in the process through which S. aureus enters the cell 12. In fact, this process is actively performed by osteoblasts, not by bacteria. FnBP binding to α5β1 integrins through Fn is the main pathway through which S. aureus enters osteoblasts via intracellular uptake. S. aureus mutants without FnBPs have difficulty become internalized in host osteoblasts 13. In a prototypical three-component FnBP-Fn-integrin interaction, FnBPs bind to Fn via a tandem β-zipper structure. The Fn-FnBPA tandem β-zipper is very mechanically stable in FnBPs-Fn-integrin structures 14. This binding triggers a conformational change in Fn, resulting in the exposure of a cryptic integrin-binding site in Fn, which in turn engages in a high-affinity interaction with the α5β1 integrin 14. Therefore, drugs that target the integrin rather than FnBPA, such as RGD peptides, are the most effective at preventing S. aureus adherence 14.

Integrins on the osteoblast surface function as signaling stimuli that lead to the endocytic uptake of the plasma membrane, which is how S. aureus enters osteoblasts 10,11. However, osteoblasts in intact bone do not usually have exposed, pathogen-accessible Fn receptors such as α5β1 integrins 10,11. Osteoblastic Fn receptors are only ubiquitously exposed on the cell surface after fracture, as these receptors facilitate cell contact and wound closure. Thus, it may be presumed that intracellular S. aureus in osteoblasts is most commonly found close to bone with an open fracture *in vivo*.

Integrins and the cytoskeleton are linked by integrin-linked kinase (ILK), and ILK interacts with the β protein cytoplasmic domain and becomes activated 15. When ILK is activated, it initiates the recruitment of focal adhesion proteins, including adaptor proteins and focal adhesion kinase (FAK) 16. Subsequently, the downstream substrates of FAK involved in the cortactin-mediated modification of the actin cytoskeleton begin to be phosphorylated. Therefore, the signaling pathway downstream of the α5β1 integrin receptor, including ILK and FAK, is important for the internalization of S. aureus.

S. aureus internalization in osteoblasts is important in bone infection. This event marks the progression of bone infection from acute to chronic, allowing bacteria to survive in cells and cause persistent infection. S. aureus can survive in osteoblasts for a long time after internalization, mainly because of two factors (Figure 1): vesicle escape and SCV formation. First, regarding vesicle escape, there is more S. aureus in macrophages than in osteoblasts, but the proportion of S. aureus that survives in osteoblasts is higher than that in macrophages. This discrepancy is caused by the inability of nonprofessional phagocytes to clear bacteria within vesicles, whereas S. aureus that invades osteoblasts can be protected in vesicles and successfully avoid proteolytic enzyme activity in the intracellular lysosome, which allows S. aureus to survive in osteoblasts for extended periods 17. The percentage of bacteria that escape depends on the infection duration; approximately 10-30% of bacteria escape after 2 hours of infection, and approximately 60-80% of bacteria escape after 8 hours, depending on the production of various toxic factors 18. At the same time, these surviving bacteria also transform into SCVs. Second, S. aureus can also acquire an SCV phenotype after cell internalization. SCVs are slow-growing bacterial subtypes with atypical colony morphology and unusual biochemical characteristics on agar plates. Compared with wild-type bacteria, SCVs have higher intracellular persistence and lower sensitivity to antibiotics, which may be associated with their lower cytotoxicity 19. Furthermore, when wild-type bacteria are converted into SCVs, the membrane potential of the bacterial membrane is reduced. This change indirectly reduces the bactericidal activity of antimicrobial agents because the transmembrane potential is critical to the uptake of positively charged particles, such as antimicrobial peptides and antibiotics 20. In addition, SCVs quickly revert to the wild-type, highly toxic, invasive phenotype upon exiting the original cells and infecting new cells, which explains why chronic osteomyelitis patients have repeated infections. In addition, the cell membrane is hydrophobic, but most antibiotics are hydrophilic, making it difficult for antibiotics to enter the cell and facilitating bacterial evasion of the activity of most antibiotics.

## Infected osteoblast destruction

S. aureus can cause extensive bone loss and bone destruction after infecting bone tissue; these characteristics are closely related to its ability to affect the balance between osteoblasts and osteoclasts. Osteoblasts become less active after S. aureus infection and begin to undergo necrosis and apoptosis. At the same time, osteoclast proliferation and activation and the bone metabolic balance are disrupted, causing inflammatory bone loss.

The activity of infected osteoblasts is reduced. Intracellular S. aureus significantly reduces osteoblast activity and eventually causes osteoblast necrosis. *In vitro* cytology studies have shown that after S. aureus infects osteoblasts, the proliferation and differentiation of osteoblasts, alkaline phosphatase activity and calcium deposition, and extracellular osteocyte matrix components (type I Cn, bone sialoprotein, osteopontin, Fn, etc.) expression are reduced. At the same time, mineralization is reduced, and the formation of new bone is inhibited 21,22.

Osteoblast necrosis in bone infection is caused by virulence factors on the surface of S. aureus, especially phenol-soluble modulins (PSMs), which are able to destroy the cell membrane of osteoblasts and cause cell lysis and death 23. Osteomyelitis caused by epidemic community-acquired methicillin-resistant S. aureus (CA-MRSA) is more severe than osteomyelitis caused by health-care-related methicillin-resistant S. aureus (HA-MRSA) infections. This difference is caused by the high expression of PSMs in CA-MRSA than HA-MRSA, which increases the ability of CA-MRSA to kill osteoblasts 24.

In addition, intracellular S. aureus can also cause osteoblast apoptosis. Apoptosis refers to autonomous and organized cell death controlled by specific genes. Cell apoptosis and necrosis involve different processes. Apoptosis is an active process rather than a passive process. It involves the activation, expression and regulation of a group of genes and is an active death process that aids in adaptation to a changing environment. Osteoblast apoptosis after intracellular S. aureus infection relies on the release of tumor necrosis factor-related apoptosis-inducing ligand (TRAIL). TRAIL interacts with the death receptors DR 4 and DR 5, which are expressed by infected osteoblasts 25, activating the programmed death pathway in osteoblasts. In this type of apoptosis, the intrinsic apoptosis pathway is activated through caspase-9 and the extrinsic apoptosis pathway is activated through caspase-8, which eventually activates caspase-3 and induces apoptosis 26-28.

Osteoblast death is critical to the deterioration associated with S. aureus osteomyelitis infection. This deterioration is multifaceted; on the one hand, the infection has resulted in reduced bone formation, and on the other hand, the re-release of viable intracellular bacteria to the extracellular space can lead to the infection of other osteoblasts, exacerbating the infection.

The proliferation and activation of osteoclasts further aggravate bone loss in osteomyelitis. Osteoblast-induced bone formation and osteoclast-induced bone resorption are always in a tightly regulated dynamic equilibrium state, which is important for maintaining the strength and integrity of the bone. The decreased proliferation and differentiation of osteoblasts infected with S. aureus promotes the proliferation and activation of osteoclasts.

**Figure 2:**
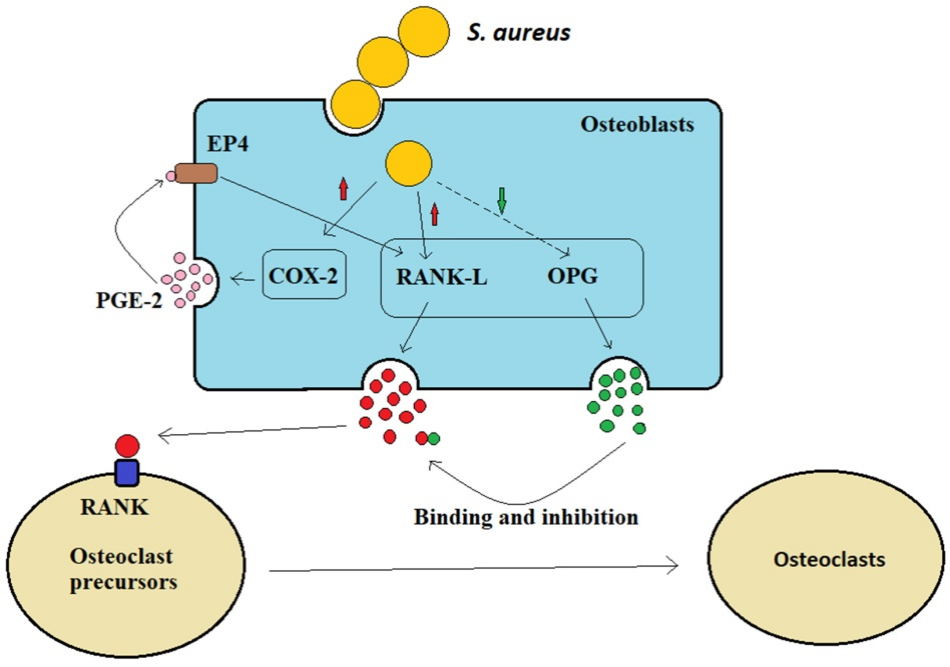
* S. aureus* indirectly promotes osteoclast formation.

Osteoblasts can secrete receptor activator of NF-kB ligand (RANK-L) and osteoprotegerin (OPG) to control bone formation (Figure 2). The interaction between osteoclast precursor cell-expressed RANK receptor and osteoblast-expressed homologous ligand RANK-L is necessary for osteoclast formation 29. Osteoclasts are multinuclear giant cells generated by the fusion of osteoprogenitor cells. The differentiation of these osteoprogenitor cells requires the activation of colony factor 1 receptor (CSF1R) by their ligand macrophage colony-stimulating factor (M-CSF or colony-stimulating factor 1/CSF1), thereby stimulating progenitor proliferation and survival. Subsequently, osteoblasts secrete RANK-L and interact with their receptor RANK on osteoclast precursor cells, inducing them to differentiate into osteoclasts 30. When osteoblasts are infected by S. aureus, they increase the production of prostaglandin E2 (PGE2), the enzymatic product of cyclooxygenase 2 (Cox-2, a hormone-like molecule), which upregulates RANK-L production via the autocrine and paracrine activation of EP4 receptors in osteoblasts. Therefore, PGE2, which is upregulated by S. aureus, acts as an additional stimulus to osteoblasts and promotes osteoclastogenesis factor generation (Figure 2). OPG is a soluble inducible receptor that targets and inhibits RANK-L to limit osteoclast generation 31 (Figure 2), whereas after S. aureus infection, osteoblasts produce less OPG and osteoclastogenesis increases. Therefore, it has been suggested that S. aureus indirectly increases osteoclastogenesis to inhibit bone formation byβ increasing the osteoblast release of RANK-L and by inhibiting OPG secretion (Figure 2).

## Treatment of infected osteoblasts

Overall, the process and mechanism of bacterial internalization are very complicated, but an accurate understanding of these processes can guide treatment. In the primary infection stage and bacterial internalization stage, recombinant CWA proteins are potential antigens that can interfere with the adhesion and internalization process of bacteria, so they can be used as a vaccine against S. aureus infection 4,32. Exogenous antimicrobial peptides (Aps)- or pro-Aps-secreted delivery drugs could potentially be used to treat bone infections. Studies have also found that an extracellular anti-inflammatory drug called serratiopeptidase reduces the invasion and internalization of S. aureus in osteoblasts 33. It also had some effects on S. aureus adhesion 34 and biofilm formation 35. However, presently, the treatment of intracellular bacteria mainly involves the use of antibiotics with high intracellular bactericidal activity.

The long-term presence of intracellular bacteria can cause the exacerbation of repeated chronic bone infection, so choosing an antibiotic that can effectively kill intracellular bacteria will be a significant milestone in osteomyelitis treatment.

Some experiments compared the intracellular bactericidal activity of several antibiotics commonly used in the clinic and found that the intracellular bactericidal activities of rifampicin, ofloxacin and clindamycin were relatively high; the activity of rifampicin 18 was especially high, and it was able to completely eradicate S. aureus in cultured osteoblasts. Although gentamicin, vancomycin and daptomycin can reduce the number of bacteria in osteoblasts, they cannot completely clear the bacteria. Some animal experiments have found that high concentrations of vancomycin can completely kill S. aureus in cells, but high concentrations of antibiotics sometimes have significant side effects 36.

Antibiotic intracellular bactericidal activity is affected by many factors, such as the ability of antibiotics to penetrate the cell membrane, the extracellular concentration of antibiotics and the pH value of the cytoplasm. These dependencies suggest that there are complementary approaches that can be used to increase the intracellular bactericidal activity of antibiotics to treat bone infections. For example, it has been pointed out that the combination of alkalinizing agents and antibiotics has a good bactericidal effect on SCVs 37. Some animal experiments have shown that antibiotics combined with targeted nanoparticles have a highly bactericidal effect on intracellular bacteria 36.

Bone infection has entered an irreversible stage by the cell destruction stage and requires surgical intervention and even amputation.

## Conclusion

Among the three stages of osteoblast infection by S. aureus, the primary stage of infection is a key to treatment. During the primary stage, the immune system is strong and bacterial levels are relatively low, which makes the infection easy to control. When S. aureus enters osteoblasts, which marks the progression of bone infection from an acute to a chronic stage, it is already difficult for the body to remove bacteria from these cells. The necrosis and apoptosis of osteoblasts (the third stage of bone infection) mark the exacerbation of bone infection and the spread of bacteria. Understanding this process of bone infection will greatly help with bone infection treatment; for example, a vaccine against the CWA proteins of S. aureus can prevent bacterial adhesion and bone infection. The choice of antibiotics with high intracellular bactericidal activity or the use of adjuvant therapy to promote the intracellular bactericidal activity of antibiotics will control repeated bone infections and represents a new method of bone infection treatment.

## Abbreviations

*S. aureus*: staphylococcus aureus
BEM: bone extracellular matrix
CWA: cell wall-anchored
FnBPs: fibronectin-binding proteins
SCV: small-colony variant
Fn: fibronectin
MSCRAMMs: microbial surface component recognizing adhesive matrix molecules
NEAT: near iron transporter
Can: collagen adhesin
Cn: collagen
Lam: laminin
ILK: integrin-linked kinase
FAK: focal adhesion kinase
PSMs: phenol-soluble modulins
CA-MRSA: community-acquired methicillin-resistant staphylococcus aureus
HA-MRSA: health-care-related methicillin-resistant staphylococcus aureus
TRAIL: tumor necrosis factor-related apoptosis-inducing ligand
RANK-L: receptor activator of NF-k B ligand
OPG: osteoprotegerin
CSF1R: colony factor 1 receptor
M-CSF: macrophage colony-stimulating factor
CSF1: colony-stimulating factor 1
PGE2: prostaglandin E2
Cox-2: cyclooxygenase 2
Aps: antimicrobial peptides

## References

[1] Saeed K, McLaren AC, Schwarz EM (2019). 2018 international consensus meeting on musculoskeletal infection: Summary from the biofilm workgroup and consensus on biofilm related musculoskeletal infections. J Orthop Res.

[2] Ferreira M, Rzhepishevska O, Grenho L (2017). Levofloxacin-loaded bone cement delivery system: Highly effective against intracellular bacteria and Staphylococcus aureus biofilms. Int J Pharm.

[3] Löffler B, Tuchscherr L, Niemann S, Peters G (2014). Staphylococcus aureus persistence in non-professional phagocytes. Int J Med Microbiol.

[4] Foster TJ, Geoghegan JA, Ganesh VK, Höök M (2014). Adhesion, invasion and evasion: the many functions of the surface proteins of Staphylococcus aureus. Nat Rev Microbiol.

[5] Patti JM, Bremell T, Krajewska-Pietrasik D (1994). The Staphylococcus aureus collagen adhesin is a virulence determinant in experimental septic arthritis. Infect Immun.

[6] Zong Y, Xu Y, Liang X (2005). A 'Collagen Hug' model for Staphylococcus aureus CNA binding to collagen. EMBO J.

[7] Herman-Bausier P, Valotteau C, Pietrocola G (2016). Mechanical Strength and Inhibition of the Staphylococcus aureus Collagen-Binding Protein Cna. MBio.

[8] Kang M, Ko YP, Liang X (2013). Collagen-binding microbial surface components recognizing adhesive matrix molecule (MSCRAMM) of Gram-positive bacteria inhibit complement activation via the classical pathway. J Biol Chem.

[9] Becker K, Heilmann C, Peters G (2014). Coagulase-negative staphylococci. Clin Microbiol Rev.

[10] Siebers MC, ter Brugge PJ, Walboomers XF, Jansen JA (2005). Integrins as linker proteins between osteoblasts and bone replacing materials. A critical review. Biomaterials.

[11] Shuaib A, Motan D, Bhattacharya P, McNabb A, Skerry TM, Lacroix D (2019). Heterogeneity in The Mechanical Properties of Integrins Determines Mechanotransduction Dynamics in Bone Osteoblasts. Sci Rep.

[12] Agerer F, Lux S, Michel A, Rohde M, Ohlsen K, Hauck CR (2005). Cellular invasion by Staphylococcus aureus reveals a functional link between focal adhesion kinase and cortactin in integrin-mediated internalisation. J Cell Sci.

[13] Ahmed S, Meghji S, Williams RJ, Henderson B, Brock JH, Nair SP (2001). Staphylococcus aureus fibronectin binding proteins are essential for internalization by osteoblasts but do not account for differences in intracellular levels of bacteria. Infect Immun.

[14] Prystopiuk V, Feuillie C, Herman-Bausier P (2018). Mechanical Forces Guiding Staphylococcus aureus Cellular Invasion. ACS Nano.

[15] Wang B, Yurecko RS, Dedhar S, Cleary PP (2006). Integrin-linked kinase is an essential link between integrins and uptake of bacterial pathogens by epithelial cells. Cell Microbiol.

[16] Boulter E, Van Obberghen-Schilling E (2006). Integrin-linked kinase and its partners: a modular platform regulating cell-matrix adhesion dynamics and cytoskeletal organization. Eur J Cell Biol.

[17] Hamza T, Li B (2014). Differential responses of osteoblasts and macrophages upon Staphylococcus aureus infection. BMC Microbiol.

[18] Valour F, Trouillet-Assant S, Riffard N (2015). Antimicrobial activity against intraosteoblastic Staphylococcus aureus. Antimicrob Agents Chemother.

[19] Tuchscherr L, Kreis CA, Hoerr V (2016). Staphylococcus aureus develops increased resistance to antibiotics by forming dynamic small colony variants during chronic osteomyelitis. J Antimicrob Chemother.

[20] Zhou K, Li C, Chen D (2018). A review on nanosystems as an effective approach against infections of Staphylococcus aureus. Int J Nanomedicine.

[21] Widaa A, Claro T, Foster TJ, O'Brien FJ, Kerrigan SW (2012). Staphylococcus aureus protein A plays a critical role in mediating bone destruction and bone loss in osteomyelitis. PLoS One.

[22] Chen Q, Hou T, Luo F, Wu X, Xie Z, Xu J (2014). Involvement of toll-like receptor 2 and pro-apoptotic signaling pathways in bone remodeling in osteomyelitis. Cell Physiol Biochem.

[23] Davido B, Saleh-Mghir A, Laurent F (2016). Phenol-Soluble Modulins Contribute to Early Sepsis Dissemination Not Late Local USA300-Osteomyelitis Severity in Rabbits. PLoS One.

[24] Rasigade JP, Trouillet-Assant S, Ferry T (2013). PSMs of hypervirulent Staphylococcus aureus act as intracellular toxins that kill infected osteoblasts. PLoS One.

[25] Young AB, Cooley ID, Chauhan VS, Marriott I (2011). Causative agents of osteomyelitis induce death domain-containing TNF-related apoptosis-inducing ligand receptor expression on osteoblasts. Bone.

[26] Jin T, Zhu YL, Li J (2013). Staphylococcal protein A, Panton-Valentine leukocidin and coagulase aggravate the bone loss and bone destruction in osteomyelitis. Cell Physiol Biochem.

[27] Alexander EH, Rivera FA, Marriott I, Anguita J, Bost KL, Hudson MC (2003). Staphylococcus aureus - induced tumor necrosis factor - related apoptosis - inducing ligand expression mediates apoptosis and caspase-8 activation in infected osteoblasts. BMC Microbiol.

[28] Claro T, Widaa A, O'Seaghdha M (2011). Staphylococcus aureus protein A binds to osteoblasts and triggers signals that weaken bone in osteomyelitis. PLoS One.

[29] Matsuo K, Irie N (2008). Osteoclast-osteoblast communication. Arch Biochem Biophys.

[30] González-Galván MC, Mosqueda-Taylor A, Bologna-Molina R, Setien-Olarra A, Marichalar-Mendia X, Aguirre-Urizar JM (2018). Evaluation of the osteoclastogenic process associated with RANK / RANK-L / OPG in odontogenic myxomas. Med Oral Patol Oral Cir Bucal.

[31] Kassem A, Lindholm C, Lerner UH (2016). Toll-Like Receptor 2 Stimulation of Osteoblasts Mediates Staphylococcus Aureus Induced Bone Resorption and Osteoclastogenesis through Enhanced RANKL. PLoS One.

[32] Schneewind O, Missiakas D (2019). Sortases, Surface Proteins, and Their Roles in Staphylococcus aureus Disease and Vaccine Development. Microbiol Spectr.

[33] Selan L, Papa R, Ermocida A (2017). Serratiopeptidase reduces the invasion of osteoblasts by Staphylococcus aureus. Int J Immunopathol Pharmacol.

[34] Papa R, Artini M, Cellini A (2013). A new anti-infective strategy to reduce the spreading of antibiotic resistance by the action on adhesion-mediated virulence factors in Staphylococcus aureus. Microb Pathog.

[35] Selan L, Papa R, Tilotta M (2015). Serratiopeptidase: a well-known metalloprotease with a new non-proteolytic activity against S. aureus biofilm. BMC Microbiol.

[36] Yang S, Han X, Yang Y (2018). Bacteria-Targeting Nanoparticles with Microenvironment-Responsive Antibiotic Release To Eliminate Intracellular Staphylococcus aureus and Associated Infection. ACS Appl Mater Interfaces.

[37] Leimer N, Rachmühl C, Palheiros Marques M (2016). Nonstable Staphylococcus aureus Small-Colony Variants Are Induced by Low pH and Sensitized to Antimicrobial Therapy by Phagolysosomal Alkalinization. J Infect Dis.

